# Metabolic and epigenetic abnormalities cause hepatic fibrogenesis in metabolic dysfunction–associated steatohepatitis model mice

**DOI:** 10.1016/j.jbc.2025.110959

**Published:** 2025-11-20

**Authors:** Atsushi Miura, Shiori Ikeda, Yuki Kono, Keigo Kawate, Takashi Hosono, Taiichiro Seki

**Affiliations:** 1 Department of Applied Life Sciences, Nihon University Graduate School of Bioresource Sciences, Fujisawa, Kanagawa, Japan; 2 Department of Chemistry and Life Science, Nihon University College of Bioresource Sciences, Fujisawa, Kanagawa, Japan; 3 Department of Bioscience, Nihon University College of Bioresource Sciences, Fujisawa, Kanagawa, Japan

**Keywords:** transcriptomics, metabolomics, histone methylation, liver metabolism, metabolic dysfunction–associated steatotic liver disease, metabolic dysfunction–associated steatohepatitis, hepatic fibrogenesis, *S*-adenosylmethionine

## Abstract

The prevalence rates of liver-related metabolic syndrome, metabolic dysfunction–associated steatotic liver disease, and the consequent metabolic dysfunction–associated steatohepatitis (MASH) are increasing worldwide. There is a well-established and reproducible choline-deficient l-amino acid–defined high-fat diet (CDAHFD)–induced MASH model, but the cause of the hepatic fibrogenesis in this model is unclear. We evaluated the phenotypic changes associated with hepatic fibrogenesis in CDAHFD-fed mice using RNA sequencing, GC–MS, and LC–MS–based metabolic analyses. A time-series liver transcriptomic analysis revealed inflammation and activated matrix remodeling within 1 week in the CDAHFD-fed mouse liver. Also, metabolomic analysis revealed increased activity in the pentose phosphate pathway, an elevated *S*-adenosylmethionine (SAM) level, and an increased SAM/*S*-adenosylhomocysteine (SAH) ratio in the CDAHFD-fed mouse liver. The increased SAM/SAH ratio positively correlated with hepatic *Tgfb1* mRNA expression. Moreover, the SAM synthesis inhibitor cycloleucine suppressed the activation of fibrogenic signaling and subsequent fibrogenesis in the liver. In the Gubra–amylin liver nonalcoholic steatohepatitis diet–fed ob/ob mouse model of MASH, increases in the SAM/SAH ratio, upregulation of fibrosis-related genes, and alterations in H3 histone methylation were observed, similar to those seen in the CDAHFD-fed MASH model. This study reveals the transcriptomic and metabolomic features of the MASH model mice and proposes a novel link between hepatic fibrogenesis and epigenetic histone modification in the context of metabolic dysfunction–associated steatotic liver disease/MASH.

The incidence of metabolic dysfunction–associated steatotic liver disease (MASLD) is increasing worldwide (1). Advanced MASLD is called metabolic dysfunction–associated steatohepatitis (MASH), which is characterized by severe inflammation and exacerbation of fibrogenesis. MASH also progresses to chronic liver disease, cirrhosis, and liver cancer. MASLD–MASH induction may be explicable by reference to the multiple parallel-hit hypothesis (2), where lipotoxicity, cytokine production, oxidative stress, and activation of cellular signaling pathways promote inflammation or *vice versa*. There are a few clinical strategies for the prevention or treatment of MASLD–MASH.

Various experimental animal models—such as genetically modified models, chemical toxicant models, and diet-induced models—have been established to investigate MASLD–MASH pathology (3, 4). There is a well-established and reproducible methionine-restricted choline-deficient l-amino acid–defined high-fat diet (CDAHFD)–induced MASH mouse model (5, 6). The CDAHFD–MASH model shows steatosis, liver inflammation, and fibrosis in a shorter time than other models (6). Decreased phosphatidylcholine synthesis and subsequently impaired excretion of lipids from the liver may cause steatosis and fibrosis (5), similar to the methionine- and choline-deficient diet-induced MASH mouse model (7). The CDAHFD induced stronger MASH responses than the methionine- and choline-deficient diet, including lipid deposition, liver injury, inflammation, bile acid overload, and hepatocyte proliferation (7). The CDAHFD–MASH model was used to explore candidate anti-MASH agents and target molecules (8, 9, 10, 11). However, these studies did not determine how CDAHFD induces liver fibrosis in rodents. To address this question, we focused on metabolic changes in the liver of CDAHFD-fed mice.

Metabolic abnormalities in lipid metabolism are a common cause of MASLD–MASH progression in human and experimental animal models (2, 3); however, carbohydrate and amino acid metabolism are also essential for metabolic homeostasis. Metabolites and metabolic enzymes regulate gene expression epigenetically (12). Metabolites, such as sugars, organic acids, amino acids, and their derivatives, modify DNA or histones in a direct manner or an indirect manner. In addition, epigenetic regulation orchestrates gene expression for fibrogenesis in the liver (13). We studied the dynamics of hydrophilic metabolites and their role in hepatic fibrogenesis.

We evaluated the relationship between metabolic abnormalities and fibrosis, and the mechanisms underlying fibrogenesis in the MASH model, using RNA-Seq, metabolomics, and conventional experiments. The CDAHFD-fed mouse liver showed a significant inflammatory response, extracellular matrix remodeling, and fibrogenic signaling during the early phase of MASH. In addition, impairment of SAM metabolism led to fibrogenic activity by altering epigenetic histone marks. To complement the CDAHFD–MASH model, we also examined a second model, the Gubra–amylin nonalcoholic steatohepatitis (GAN) diet–fed ob/ob mouse model, a modification of the traditional *trans*–fatty acid–rich amylin liver nonalcoholic steatohepatitis diet in which Primex shortening is replaced by an equal amount of palm oil (14). Taken together, these findings provide new mechanistic insight into MASH pathogenesis, revealing that dysregulation of methionine metabolism and histone methylation constitutes a common pathway driving hepatic fibrogenesis across distinct experimental models.

## Results

### Hepatic steatosis, liver injury, and fibrogenic signaling in the CDAHFD–MASH mouse model

**Figure 1:**
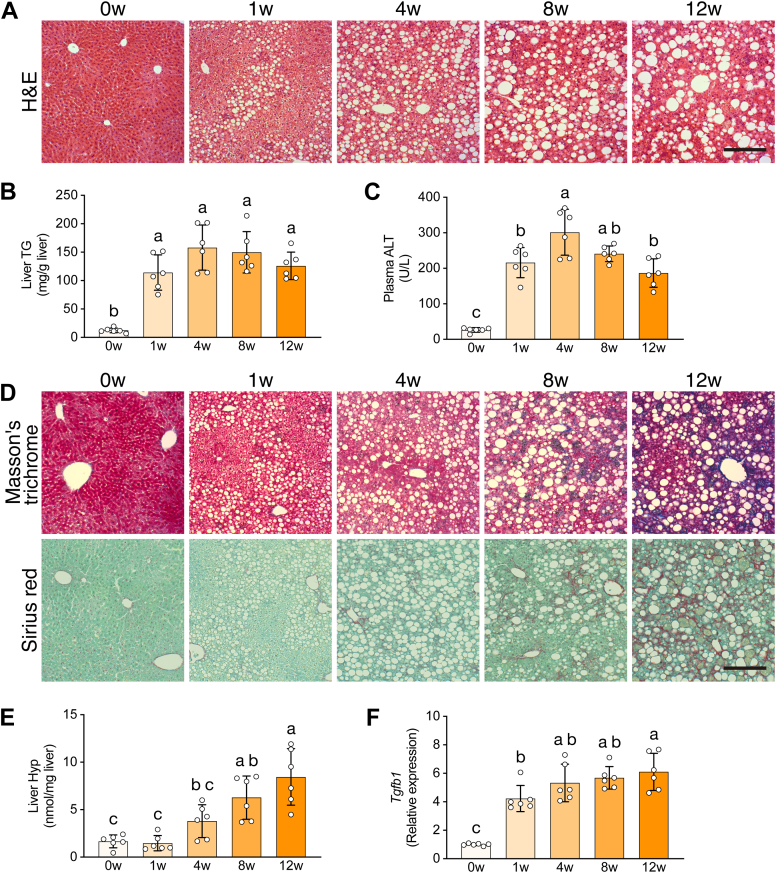
**Steatosis, fibrosis, and liver injury in the choline-deficient L-amino acid–defined high-fat diet (CDAHFD–induced metabolic dysfunction–associated steatohepatitis (MASH) mouse model**. *A*, representative images of liver sections stained with H&E. *B*, hepatic triglyceride (TG) content. *C*, plasma alanine aminotransferase (ALT) activity. *D*, representative images of liver sections stained with Masson’s trichrome and Sirius red. *E*, hepatic hydroxyproline (Hyp) content. *F*, hepatic expression of *Tgfb1*. Data are means ± SD (n = 6). Different letters indicate significant differences by Tukey’s test, α = 0.05.

To characterize the CDAHFD-induced MASH mouse model, we conducted a time-series experiment in which mice were given a CDAHFD for 12 weeks. We confirmed the liver histology of the CDAHFD-fed mice by H&E staining (Fig. 1*A*). We observed lipid droplets and hepatocellular hypertrophy in the CDAHFD-fed mouse liver at 1 week. These histological features progressed over time. Also, the hepatic triglyceride (TG) content increased markedly at 1 week (Fig. 1*B*). The hepatic cholesterol (CHO) content also increased gradually (Fig. S1*A*). By contrast, the CDAHFD did not affect the plasma TG or CHO levels (Fig. S1*B*). To assess liver injury, we measured plasma levels of the liver injury markers alanine aminotransferase (ALT) and aspartate aminotransferase (AST) (Figs. 1*C*, S1*C*). Plasma ALT and AST levels were higher in CDAHFD-fed mice than in control mice at 0 weeks. Therefore, the CDAHFD–MASH model mice showed hepatic steatosis and liver injury at an early phase of experimental MASH.

To evaluate hepatic fibrogenesis, we histologically analyzed the CDAHFD-fed mouse liver by Masson’s trichrome and Sirius red staining, which distinguish deposited collagen fibers and other materials in liver tissue sections. Marked collagen deposition was observed at 8 and 12 weeks (Fig. 1*D*, S1*D*). In addition, we measured the hepatic hydroxyproline (Hyp) content, which reflects the hepatic collagen content. The CDAHFD-fed mouse liver showed a high Hyp level at 4 to 12 weeks (Fig. 1*E*). To verify the activation of fibrogenic signaling, we measured the mRNA expression of fibrogenesis-related genes by quantitative PCR (qPCR). The expression level of the fibrogenic master regulator *Tgfb1* increased dramatically in the CDAHFD-fed mouse liver at 1 week, and the increase was maintained until the end of the experiment (Fig. 1*F*). The levels of the fibrogenic proteins, *Col1a1*, *Col3a1*, and *Acta2*, also increased substantially in the CDAHFD-fed mouse liver at 1 week (Fig. S1*E*). The levels of the fibrogenic regulators, *Ctgf* and *Bambi*, gradually increased over time (Fig. S1*E*). Therefore, the CDAHFD led to fibrogenic activity in the liver at an early phase of experimental MASH. Taken together, the aforementioned findings demonstrate that CDAHFD–MASH model mice show early steatohepatitis, liver fibrosis, and fibrogenic signaling in the liver.

### Increased macrophage recruitment and matrix remodeling in CDAHFD-fed mouse liver

**Figure 2:**
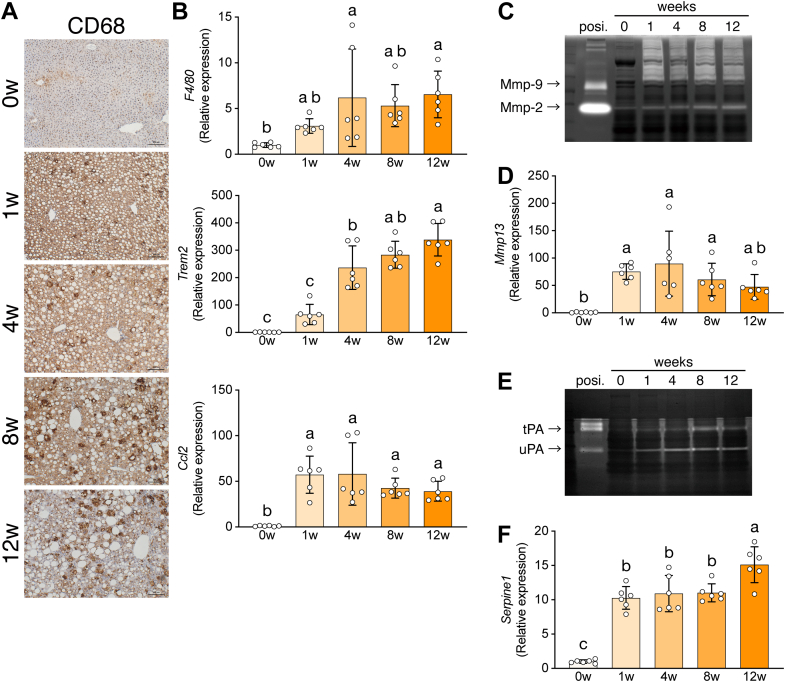
**Macrophage recruitment and extracellular matrix degradation in the CDAHFD-fed mouse liver**. *A*, representative images of liver sections subjected to CD68 immunostaining. *B*, hepatic expression of *F4/80 (Adgre1)*, *Trem2*, and *Ccl2*. *C*, hepatic matrix metalloproteinase (MMP)-2 and MMP-9 activity. *D*, hepatic expression of *Mmp13* mRNA. *E*, hepatic tPA and uPA activity. *F*, hepatic expression of *Serpine1 (plasminogen activator inhibitor[PAI]-1)*. Data are means ± SD (n = 6). Different letters indicate significant differences by Tukey’s test, α = 0.05. CDAHFD, choline-deficient L-amino acid–defined high-fat diet; tPA, tissue-type plasminogen activator; uPA, urokinase-type plasminogen activator.

Inflammatory processes, such as macrophage recruitment and cytokine production, promote MASH (4). To determine the inflammatory status of the CDAHFD-fed mouse liver, we performed CD68 immunostaining to visualize the distribution of phagocytic macrophages (Fig. 2*A*). CD68-positive cells were recruited and aggregated in the CDAHFD-fed mouse liver at 1 week. To confirm the histological observations, we analyzed the expression of inflammation-associated genes, including macrophage markers and cytokines, by qPCR. Consistent with the histological results, the mRNA levels of the pan-macrophage marker *F4/80 (Adgre1)* and MASH-associated macrophage markers, *Trem2* and *Gpnmb*, increased gradually over 12 weeks (Figs. 2*B*, S1*F*). Also, the expression levels of the macrophage-mediated inflammatory cytokines, *Ccl2* and *Tnfa*, were higher in the CDAHFD-fed mouse liver compared with the control at 0 weeks (Figs. 2*B*, S1*G*). In addition, *Il6* mRNA expression slightly increased at 8 to 12 weeks (Fig. S1*G*). These results indicate that the CDAHFD promoted macrophage recruitment and activation in the liver during the early phase of experimental MASH.

Matrix remodeling regulates liver fibrogenesis (15). Matrix metalloproteinases (MMPs) degrade pericellular matrix components, including collagen, elastin, and fibronectin. To assess the effect of the CDAHFD on matrix remodeling in the liver, we analyzed the gene expression and enzymatic activity of MMPs in the mouse liver by qPCR and gelatin zymography. MMP-2 activity increased over time in the CDAHFD-fed mouse liver (Fig. 2*C*). The mRNA expression of *Mmp2* and *Timp1* also increased in the liver (Fig. S1*H*). Macrophages produce MMP-9 and MMP-13 during matrix remodeling. The CDAHFD rapidly induced enzymatic activity and mRNA expression of MMP-9 at 1 week (Figs. 2*C*, S1*H*). The gene expression of *Mmp13* also increased in the CDAHFD-fed mouse liver (Fig. 2*D*). Tissue- and urokinase-type plasminogen activators (PAs) activate MMPs during matrix remodeling *via* proteolytic degradation on the macrophage surface (16, 17). Therefore, we analyzed the gene expression and enzymatic activity of PAs in the mouse liver by qPCR and fibrinogen zymography (Figs. 2*E*, S1*I*). CDAHFD induced gene expression and enzymatic activity of PAs in the liver. The gene expression of *Serpine1*, encoding a physiological inhibitor of PAs, increased in the CDAHFD-fed mouse liver at 1 week (Fig. 2*F*). Therefore, the CDAHFD promoted extracellular matrix remodeling in the mouse liver, mediated in part by macrophages. Therefore, the CDAHFD–MASH model mice show increased macrophage recruitment and matrix remodeling during the early phase of experimental MASH. Moreover, these processes progressed in parallel with fibrogenesis.

### MASH transcriptomic signatures and metabolic abnormalities in CDAHFD-fed mouse liver

**Figure 3:**
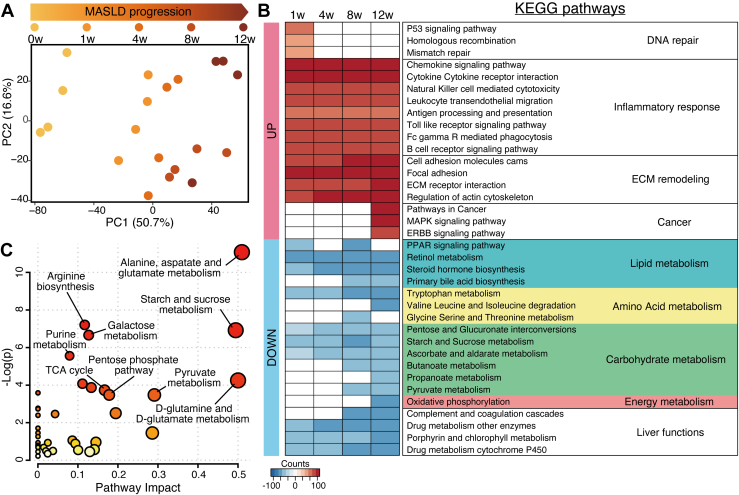
**Transcriptomic and metabolomic characterization of the CDAHFD-fed mouse liver**. *A*, principal component analysis (PCA) of the liver transcriptome by RNA-Seq. *B*, enrichment analysis of the liver transcriptome by RNA-Seq. See Figure S2*B* for statistical criteria. *C*, pathway analysis of the data obtained by GC–MS-based untargeted metabolomic analysis. See Figure S3*B* for statistical criteria. CDAHFD, choline-deficient L-amino acid–defined high-fat diet.

To characterize further the phenotypic changes in the CDAHFD-fed mouse liver, we conducted transcriptomic and metabolomic analyses (RNA-Seq and GC–MS-based untargeted analysis, respectively) as unbiased approaches. Principal component analysis showed that the liver transcriptome changed over time (Figs. 3*A*, S2*A*). To identify the biological processes promoted or suppressed in parallel with the progression of MASH, we performed Gene Ontology and pathway analyses (Figs. 3*B*, S2, *B* and *C*). We identified differentially expressed genes based on specific statistical criteria (Fig. S2*B*) as well as unique and overlapping Gene Ontology terms (Figs. 3*B*, S2*C*). The DNA repair pathways were transiently enhanced at 1 week. The pathways of inflammatory response and extracellular matrix remodeling were continuously activated during CDAHFD feeding, and the activity in these pathways increased over time. In addition, activity in cancer-associated pathways, such as mitogen-activated protein kinase and ERBB (erythroblastic leukemia viral oncogene homolog) signaling, was enhanced at 12 weeks. By contrast, several nutrient/energy metabolic pathways were suppressed in the CDAHFD-fed mice. Notably, oxidative phosphorylation was suppressed at 12 weeks. In addition, the CDAHFD suppressed specialized functions of the liver, such as detoxification, coagulation, steroid biosynthesis, and heme metabolism. Using a heatmap, we visualized a set of genes from a human liver MASH transcriptomic study (18), which confirmed that human MASH–associated genes changed over time in the CDAHFD-fed mouse liver (Fig. S2*D*).

Next, we identified a set of metabolites that changed over time in parallel with the progression of MASH by multivariate analysis (Fig. S3, *A* and *B*). We also conducted an enrichment analysis to identify metabolic pathways affected by the CDAHFD (Fig. 3*C*). The CDAHFD affected amino acid, carbohydrate, nucleotide, and energy metabolism. Notably, the CDAHFD also affected some metabolic pathways, including starch/sucrose metabolism, pentose phosphate pathway, and pyruvate metabolism, consistent with the results of transcriptomic analysis. To summarize and visualize the data, we mapped the genes and metabolites that changed over time, deriving manually curated metabolic pathways (Fig. S3*C*). The map revealed that the pentose phosphate pathway was upregulated during the progression of MASH. Regarding the tricarboxylic acid cycle, the results were inconsistent between the transcriptome and metabolome in the liver. To verify the pathway mapping, we analyzed the mRNA expression of *G6pdx*, the rate-limiting enzyme of the pentose phosphate pathway, and performed a correlation analysis between *G6pdx* expression and the d-ribulose 5-phosphate level in the liver (Fig. S3*D*). The expression of *G6pdx* increased over time and was positively correlated with that of d-ribulose 5-phosphate in the liver. Therefore, the CDAHFD-fed mouse liver shows the characteristic MASH transcriptomic signatures and metabolic abnormalities.

### Link between abnormalities in methionine–SAM–cysteine metabolism and fibrogenesis in CDAHFD–MASH model mice

**Figure 4:**
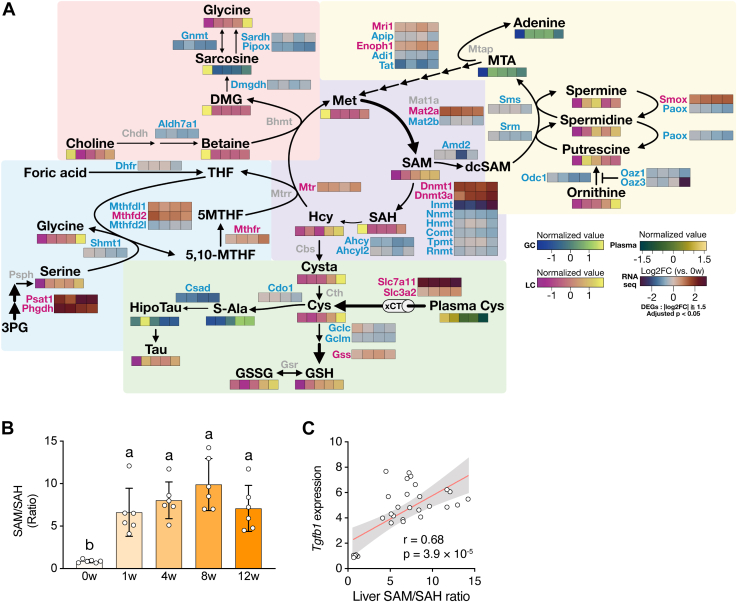
**Abnormality in methionine-related metabolic pathways in the CDAHFD-fed mouse liver**. *A*, curated metabolic pathway. Each colored block indicates the standardized gene count or metabolite abundance. *Red*, upregulated; *blue*, downregulated; and *gray*, not significant. *B*, hepatic SAM/SAH ratio. Data are means ± SD (n = 6). Different letters indicate significant differences by Tukey’s test, α = 0.05. *C*, correlation analysis of the hepatic SAM/SAH ratio and *Tgfb1* expression; *r*, Pearson’s correlation coefficient. CDAHFD, choline-deficient L-amino acid–defined high-fat diet.

We next explored the metabolic changes that cause fibrogenesis in the liver. Because of the composition of the CDAHFD, we focused on choline- and methionine-related metabolic pathways. To identify candidate metabolites and metabolic hubs in the liver, we performed targeted metabolic profiling by LC–MS/MS and mapped the data to the manually curated pathways (Fig. 4*A*). The cysteine content increased over time in the CDAHFD-fed mouse liver (Fig. S4*A*). In addition, the contents of cystathionine and glutathione, which are synthesized from cysteine, increased in the CDAHFD-fed mouse liver (Fig. S4*A*). The CDAHFD did not affect the choline content; whereas the betaine, dimethylglycine, and methionine contents decreased in the CDAHFD-fed mouse liver (Fig. S4, *B* and *C*). To confirm these results, we performed plasma amino acid profiling (Fig. 4*A*, S4*D*). Notably, the plasma levels of the sulfur-containing amino acids, methionine, taurine, and cysteine, decreased over time in the CDAHFD-fed mice (Fig. S4, *D* and *E*). In addition, we observed increased gene expression of *Slc7a11* and *Slc3a2*, which comprise the cysteine transporter complex xCT, in the CDAHFD-fed mouse liver (Fig. 4*A*). The hepatic cysteine content correlated with the plasma cysteine level and gene expression level of *Slc7a11* (Fig. S4*F*); therefore, our data suggest that the liver incorporates cysteine from plasma.

Unexpectedly, the SAM content increased over time in the CDAHFD-fed mouse liver (Fig. S4*G*). By contrast, the content of SAH, which is converted from SAM, in the CDAHFD-fed mouse liver was lower than in the mouse liver at 0 weeks, consistent with the hepatic methionine level (Fig. S4, *C* and *G*). SAM is the methylation donor for many methyl acceptors, such as DNA, histone, and metabolites (19). The SAM/SAH ratio reflects the cellular methylation potential (20). Therefore, we focused on SAM and its metabolite SAH in the liver. The CDAHFD-fed mouse liver had a higher SAM/SAH ratio than the control at 0 weeks (Fig. 4*B*). Importantly, the liver SAM and SAH levels, and the SAM/SAH ratio, correlated with the gene expression of *Tgfb1* in the liver (Fig. 4*C*, S4*H*). Therefore, abnormalities in SAM metabolism may cause fibrogenesis in the CDAHFD-fed mouse liver.

### SAM metabolism abnormalities activate fibrogenic signaling and MASH pathology in CDAHFD-fed mice

**Figure 5:**
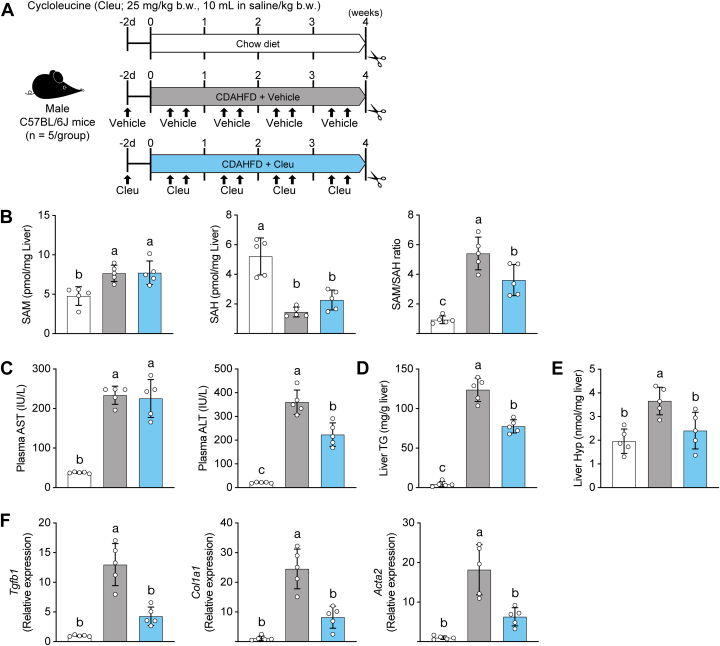
**Effect of Cleu on SAM metabolism, liver injury, steatosis, and fibrosis in the CDAHFD-fed mouse liver**. *A*, SAM synthesis inhibition experiment. *B*, hepatic SAM, SAH content, and SAM/SAH ratio. *C*, plasma AST and ALT activity. *D*, hepatic TG content. *E*, hepatic Hyp content. *F*, hepatic expression of *Tgfb1*, *Col1a1*, and *Acta2*. Data are means ± SD (n = 5). Different letters indicate significant differences by Tukey’s test, α = 0.05. ALT, alanine aminotransferase; AST, aspartate aminotransferase; CDAHFD, choline-deficient L-amino acid–defined high-fat diet; Cleu, cycloleucine; Hyp, hydroxyproline; TG, triglyceride.

**Figure 6:**
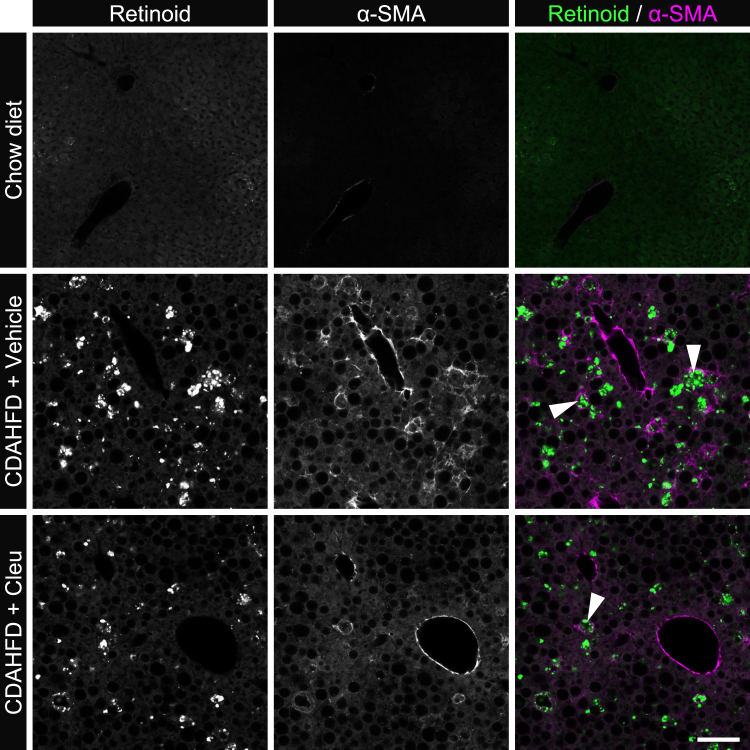
**Effect of Cleu on hepatic histology in CDAHFD-fed mice**. Representative images of liver sections subjected to α-SMA immunostaining and autofluorescence of retinoid signals. *Arrowheads*: α-SMA- and retinoid-positive cells. The scale bar represents 100 μm. CDAHFD, choline-deficient L-amino acid–defined high-fat diet; Cleu, cycloleucine; α-SMA, α-smooth muscle actin.

Based on our observations, we hypothesized that inhibition of SAM synthesis suppresses fibrogenic signaling in the liver of CDAHFD-fed mice. To test this hypothesis, we administered the methionine adenosyltransferase (MAT) inhibitor cycloleucine (1-aminocyclopentane-1-carboxylic acid; hereafter referred to as Cleu) to CDAHFD-fed mice to inhibit SAM synthesis (Fig.5*A*) (21, 22). First, we confirmed that Cleu affected SAM metabolism in the liver by targeted metabolic profiling. Cleu treatment significantly reduced the hepatic SAM/SAH ratio compared with vehicle treatment (Fig. 5*B*). In CDAHFD-fed mice, serum AST and ALT activities were elevated; however, Cleu administration significantly reduced ALT activity (Fig. 5*C*). Moreover, hepatic TG accumulation and hepatic collagen content determined by quantification of Hyp were markedly increased in CDAHFD-fed mice, whereas Cleu administration ameliorated hepatic steatosis and fibrosis (Fig. 5, *D* and *E*). To evaluate the activation of fibrogenic signaling, we analyzed the mRNA expression of *Tgfb1*, *Col1a1*, and *Acta2* by qPCR (Fig. 5*F*). Cleu treatment markedly decreased the expression levels of these genes. Hepatic stellate cells (HSCs) are central players in liver fibrosis development and progression. Upon liver injury, quiescent HSCs transdifferentiate into an activated, myofibroblast-like phenotype that drives excessive collagen deposition. As metabolic liver fibrosis progresses, HSCs display a distinctive phenotype of hypertrophied, retinoid-laden cells that expand spatiotemporally in parallel with collagen deposition within fibrotic regions. Consequently, retinoid autofluorescence can be observed within these cells (23). In CDAHFD-fed mice, cells positive for both retinoid autofluorescence and the activated HSC marker, α-smooth muscle actin (α-SMA), were readily detected, whereas Cleu administration markedly attenuated these HSC-associated signals (Fig. 6).

**Figure 7:**
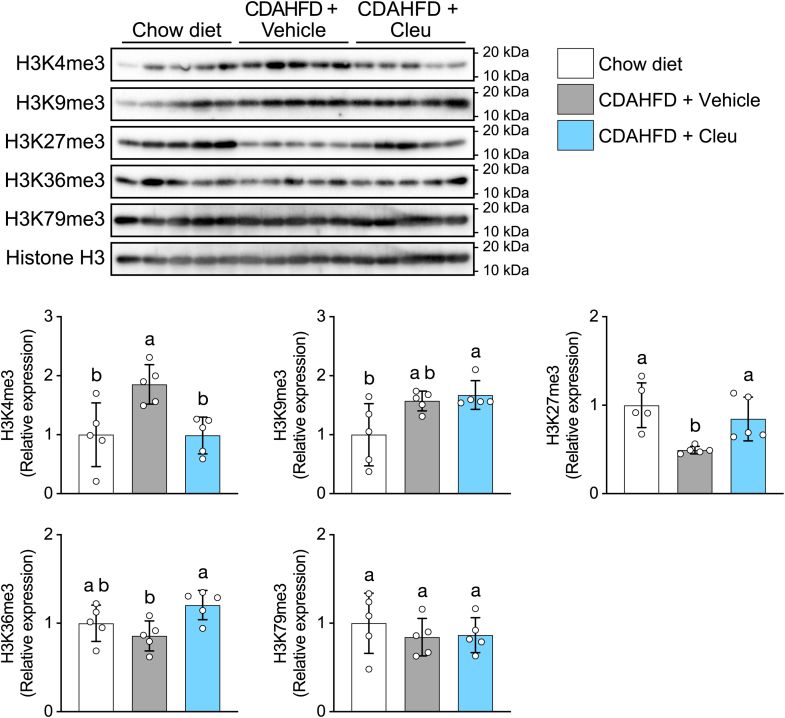
**Effect of Cleu on histone modification in the CDAHFD-fed mouse liver**. Histone modification analysis of H3K4me3, H3K9me3, H3K27me3, H3K36me3, and H3K79me3. Total histone H3 was used as the loading controls. Densitometric analysis was performed using ImageJ software. Data are means ± SD (n = 5). Different letters indicate significant differences by Tukey’s test, α = 0.05. CDAHFD, choline-deficient L-amino acid–defined high-fat diet; Cleu, cycloleucine.

To clarify the mechanisms underlying SAM-mediated activation of fibrogenic signaling, we focused on histone methylation. SAM serves as a methyl donor that promotes histone methylation under various conditions (18), and histone methylation has been reported to be influenced by MASLD–MASH (24, 25, 26). Therefore, we assessed hepatic levels of the histone marks, H3K4me3, H3K9me3, H3K27me3, H3K36me3, and H3K79me3, by Western blotting (Fig. 7). CDAHFD-fed mouse livers exhibited increased levels of histones, H3K4me3 and H3K9me3, and decreased H3K27me3. Cleu administration reversed the CDAHFD-induced increase in H3K4me3 and decrease in H3K27me3. Histone methylation–dependent transcriptional regulation has been reported to contribute to the progression of liver fibrosis. Therefore, these findings suggest that dysregulated SAM metabolism promotes fibrogenic signaling and fibrosis in the liver, at least in part, through histone H3 modifications in the CDAHFD-induced MASH mouse model.

### SAM metabolism and fibrogenesis in GAN diet–fed ob/ob mice

**Figure 8:**
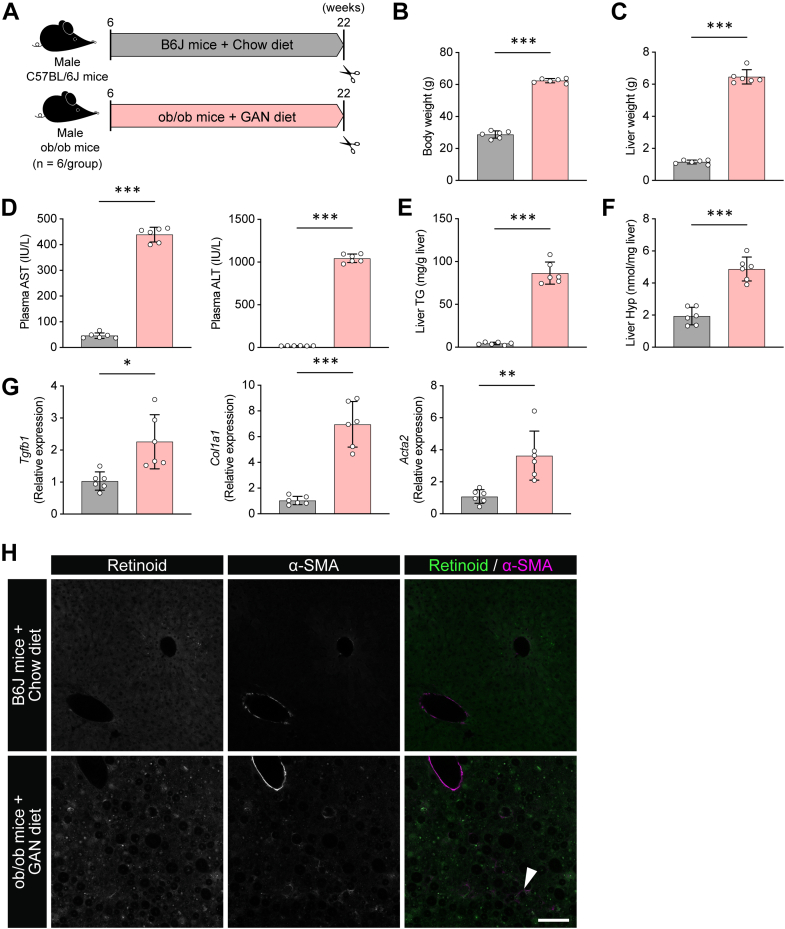
**Hepatic steatosis and fibrosis in GAN diet–fed ob/ob mice**. *A*, GAN diet–fed ob/ob mice experiment. *B*, body weight. *C*, liver weight. *D*, plasma AST and ALT activity. *E*, hepatic TG content. *F*, hepatic Hyp content. *G*, hepatic expression of *Tgfb1*, *Col1a1*, and *Acta2*. Data are means ± SD (n = 6). ∗*p* < 0.05, ∗∗*p* < 0.01, and ∗∗∗*p* < 0.001 by Welch’s *t* test. *H*, representative images of liver sections subjected to α-SMA immunostaining and autofluorescence of retinoid signal. The scale bar represents 100 μm. ALT, alanine aminotransferase; AST, aspartate aminotransferase; GAN, Gubra–amylin nonalcoholic steatohepatitis; Hyp, hydroxyproline; α-SMA, α-smooth muscle actin; TG, triglyceride.

Finally, we generated an MASH model by feeding leptin-deficient (ob/ob) mice the GAN diet for 16 weeks (Fig. 8*A*). Control C57BL/6J (B6J) mice received standard chow for the same duration. This MASH model is characterized by significant weight gain and metabolic dysfunction driven by excessive saturated fat, fructose, and CHO. Compared with chow-B6J mice, GAN diet–fed ob/ob mice increased body and liver weight (Fig. 8, *B* and *C*). Plasma liver injury markers (AST and ALT) and hepatic TG content were increased in GAN diet–fed mice (Fig. 8, *D* and *E*). Hepatic collagen content determined by quantification of Hyp and the expression of fibrogenic marker genes (*Tgfb1*, *Acta2*, and *Col1a1*) were also markedly increased in GAN diet–fed mice (Fig. 8, *F* and *G*). Histological analysis identified retinoid autofluorescence and α-SMA positivity in HSCs (Fig. 8*H*). Collectively, these data indicate that 16 weeks of GAN diet feeding in ob/ob mice induce hepatic steatosis and fibrosis.

**Figure 9:**
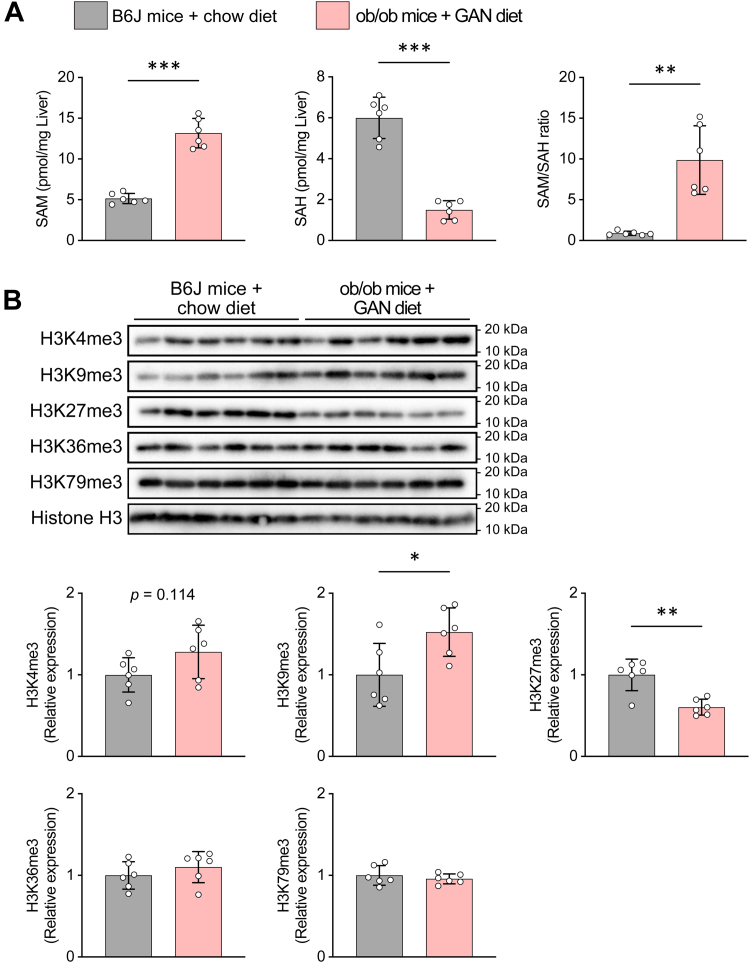
**Hepatic SAM metabolism and histone modification profiles in GAN diet–fed ob/ob mice**. *A*, hepatic SAM content, SAH content, and SAM/SAH ratio. *B*, histone modification analysis of H3K4me3, H3K9me3, H3K27me3, H3K36me3, and H3K79me3. Total histone H3 was used as the loading controls. Densitometric analysis was performed using ImageJ software. Data are means ± SD (n = 6). ∗*p* < 0.05, ∗∗*p* < 0.01, and ∗∗∗*p* < 0.001 by Welch’s *t* test. GAN, Gubra–amylin nonalcoholic steatohepatitis.

In the CDAHFD-fed MASH model, in which dietary choline and methionine are restricted, abnormalities in methionine metabolism were accompanied by an increased SAM/SAH ratio, elevations in H3K4me3 and H3K9me3, and a decrease in H3K27me3. We therefore evaluated the SAM/SAH ratio and histone H3 methylation in GAN diet–fed ob/ob mice as well. An increased SAM concentration together with a decreased SAH concentration yielded an elevated SAM/SAH ratio (Fig. 9*A*). Consistent with the CDAHFD–MASH model, histone H3 methylation changes in GAN diet–fed ob/ob mice showed a trend toward increased H3K4me3, increased H3K9me3, and decreased H3K27me3 (Fig. 9*B*). Together, these findings indicate that, even in an MASH model distinct from CDAHFD, hepatic fibrosis progresses *via* an elevated SAM/SAH ratio accompanied by alterations in histone methylation.

## Discussion

We characterized the phenotypic changes of the liver in CDAHFD–MASH model mice. We observed marked lipid accumulation in the CDAHFD-fed mouse liver within 1 week. Several articles have similarly reported rapid steatosis at 1 week after CDAHFD feeding (5, 6). Furthermore, we detected upregulated genes related to inflammation and matrix remodeling at 1 week. We also observed macrophage recruitment and activation of matrix-degrading enzymes in the CDAHFD-fed mouse liver at 1 week. Therefore, the CDAHFD rapidly enhanced hepatic fibrogenesis. A longitudinal transcriptome analysis showed upregulated inflammatory and fibrotic gene signatures after 2 weeks in a high-fat, high-fructose, and high-CHO diet–fed ob/ob mouse model (10). Therefore, the CDAHFD–MASH model recapitulates the early transcriptomic features of MASH. Abe *et al*. (10) reported that the CDAHFD–MASH model does not replicate human MASH because the CDAHFD does not induce insulin resistance and weight gain. However, the transcriptome of the CDAHFD–MASH model shares some signature genes with the human MASH transcriptome. Therefore, we suggest that the CDAHFD–MASH is useful for MASH research.

The CDAHFD-fed mouse liver has characteristic features of carbohydrate and amino acid metabolism. The CDAHFD decreases the plasma insulin level in C57BL/6J mice (10). Also, our data indicated abnormalities in carbohydrate metabolic pathways, such as the starch/sucrose metabolic pathway and the pentose phosphate pathway. Specifically, the model shows abnormal hepatic glucose catabolism and impaired energy production, which may affect systemic glucose metabolism. Notably, the CDAHFD-fed mouse liver showed increased the pentose phosphate pathway activity. The pentose phosphate pathway produces NADPH for glutathione reduction and fatty acid synthesis (27). Jin *et al*. (28) reported that the pentose phosphate pathway activity reflects reductive TG synthesis, but not oxidative stress, in the fatty liver of obese rats. In this study, the glutathione level, which reflects oxidative stress, did not decrease in the CDAHFD-fed mouse liver. Also, we found evidence that the liver incorporates cysteine from plasma in experimental MASH. Our findings suggest that the CDAHFD-fed mouse liver maintains its reductive potential against oxidative stress. This suggests that the increased pentose phosphate pathway activity reflects abnormal lipogenesis, but not oxidative stress, in the CDAHFD-fed mouse liver under lipid overload conditions. Although we did not identify a contribution of pentose phosphate pathway activity to steatosis, inflammation, or fibrosis, our data suggest that pentose phosphate pathway is associated with inflammation and the consequent fibrogenesis in MASH.

This study demonstrates that, in two mechanistically distinct MASH models, CDAHFD and GAN diet–fed ob/ob mice, there is a common pattern comprising elevation of the intrahepatic SAM/SAH ratio, a shift in histone H3 marks (increased H3K4me3, H3K9me3, and decreased H3K27me3), and concomitant transcriptional activation of fibrosis-related gene sets. Furthermore, pharmacological reduction of the SAM/SAH ratio by administering Cleu, an inhibitor of MAT, led to a decrease in both steatosis and hepatic fibrosis. Collectively, these findings support a pathogenic cascade in which the accumulation of SAM leads to epigenetic remodeling, which in turn activates HSCs, thereby contributing to fibrosis and, ultimately, MASH progression.

Excessive hepatic SAM/SAH ratios have been associated with an elevated risk of liver disease (29). In instances of hepatic SAM reduction resulting from MAT deficiency or disruption of the phosphatidylethanolamine *N*-methyltransferase pathway, impaired phosphatidylcholine synthesis compromises lipid export, consequently leading to steatosis (30). In contrast, in cases of glycine *N*-methyltransferase deficiency, the accumulation of SAM leads to a manifestation of an MASH-like phenotype, progressing from steatosis to fibrosis. The elevated SAM/SAH ratio observed in this study is consistent with the latter mechanism and aligns with the transcriptional upregulation of fibrotic genes, such as *Tgfb1*, *Acta2*, and *Col1a1*, mediated by increased H3K4me3 and decreased H3K27me3 in HSCs (31, 32). Concurrently, elevated H3K4me3 levels at the Tgfb1 locus have been documented in human liver disease specimens (33).

Despite the frequent criticism of CDAHFD as a nonphysiological model because of its restricted choline and methionine content, it is noteworthy that a similar elevation in the SAM/SAH ratio and a comparable epigenetic shift were reproduced in the GAN diet–fed ob/ob model, which is driven by excess saturated fatty acid, fructose, and CHO. This finding indicates a shared pathological mechanism, specifically the dysregulation of the methionine cycle under conditions of lipid and carbohydrate overload. This observation refutes the hypothesis that the phenomenon is exclusive to methionine and choline restriction. The following potential mechanisms have been identified: The process of SAM synthesis is known to be activated under certain conditions. For example, an increased Mat2a/Mat2b ratio has been observed in some cases. In addition, impaired SAM consumption can result from reduced expression or function of glycine *N*-methyltransferase/nicotinamide *N*-methyltransferase. Finally, diminished SAH clearance can be caused by relative insufficiency in one-carbon metabolism (folate, vitamin B_12_, and vitamin B_6_) and/or the choline–betaine pathway.

With regard to histone modifications, increased H3K4me3 and H3K9me3 and decreased H3K27me3 accompany HSC activation, and these modifications have been implicated in the transcriptional activation of fibrosis-related genes (31, 32, 34). It has been reported that there is additional involvement of methylation enzymes, including H3K4me3 deposition by ASH1-like histone lysine methyltransferase and regulation of epithelial–mesenchymal transition *via* H3K9me3 demethylation by lysine-specific demethylase 4C (35, 36). Given that SAM is a ubiquitous methyl donor for DNA, histones, phospholipids, and RNA, the locus-specific changes in H3K4me3, H3K9me3, and H3K27me3 observed here support the notion that SAM-coupled epigenetic plasticity is a tractable therapeutic target in MASH. There are several limitations to this study. First, the causal sequence linking an elevated SAM/SAH ratio to histone methylation changes and fibrosis cannot be demonstrated definitively by pharmacological intervention with Cleu alone. Definitive tests of pathway necessity will require liver-specific genetic perturbation of histone methyltransferases and demethylases, coupled with functional readouts; these experiments are planned as the next step. Second, we did not perform proteome-wide LC–MS/MS profiling of histone methylation or chromatin immunoprecipitation sequencing. To relate the elevated SAM/SAH ratio to chromatin changes, we relied on immunoblotting of selected marks. Establishing the generality and locus specificity of these effects will require future studies incorporating validated LC–MS/MS profiling together with genome-wide assays.

In summary, we characterized the features of the CDAHFD–MASH model and the GAN diet–ob/ob mouse MASH model. In livers of CDAHFD-fed mice, abnormalities were observed in carbohydrate and amino acid metabolism, including the pentose phosphate pathway and methionine–cysteine metabolism. Furthermore, elevation of the SAM/SAH ratio, together with aberrant epigenetic regulation, promoted liver fibrosis, whereas Cleu mitigated fibrosis by normalizing these changes. Similarly, in GAN diet–ob/ob mice, liver fibrosis was induced *via* methionine metabolism–dependent abnormalities in epigenetic regulation, as in the CDAHFD model. Collectively, these findings suggest that restoring epigenetic regulation *via* the SAM/SAH ratio is a promising target for the prevention and treatment of MASH. Future studies clarifying the relationship between the SAM/SAH ratio and epigenetic dysregulation in human MASH specimens may facilitate the development of antifibrotic strategies that do not rely on pharmacotherapy.

## Experimental procedures

### Mice

All experiments using laboratory animals were approved by the Nihon University Animal Care and Use Committee (approval nos.: AP17B107-2, AP20BRS048-1, AP20BRS059-1, AP24BRS112-1, and AP25BRS001-1). The mice were housed under a constant light–dark cycle (12:12 h) and a controlled temperature (22°C) during the acclimation and experimental periods.

For the time-series experiment, male C57BL/6J (B6J) mice (10 weeks old; n = 6 per group) were fed the choline-deficient l-amino acid–defined (0.1% methionine) 60% kcal high-fat diet (CDAHFD; catalog no.: A06071302; Research Diets) for 1, 4, 8, and 12 weeks. The age- and sex-matched acclimatized mice fed a standard chow diet (CRF-1; Oriental Yeast) were assigned to the control group (0 weeks).

For the SAM synthesis inhibition experiment, male B6J mice (10 weeks old; n = 5 per group) were fed the CDAHFD for 4 weeks. Two days before the start of CDAHFD feeding, the mice were intraperitoneally administered Cleu (catalog no.: A1063; Tokyo Chemical Industry; 25 mg/kg bodyweight, 10 ml/kg bodyweight); this was repeated twice weekly thereafter for 4 weeks. Cleu was dissolved in sterile saline (Otsuka Pharmaceutical Factory), and sterile saline was used as the vehicle.

For an additional MASH model, male B6.Cg-Lep^ob^/J (ob/ob) mice (6 weeks old; n = 6 per group) were fed the GAN diet (catalog no.: D09100310; Research Diets) for 16 weeks (40 kcal% fat [46% saturated fatty acids by weight], 22% fructose, 10% sucrose, 2% CHO) (14). Age- and sex-matched, acclimatized B6J mice fed a standard chow diet (CRF-1) served as controls.

### Plasma and liver analysis

The mice were euthanized by carbon dioxide inhalation, and tissues were collected and snap frozen in liquid nitrogen. EDTA was added to blood collected by cardiac puncture, and plasma was prepared by centrifugation (2000 × *g*, 15 min, 4 °C). The tissue and plasma samples were stored at −80 °C until analysis. For histological analysis, the collected fresh liver was fixed in 4% paraformaldehyde in phosphate buffer at 4 °C.

### Histological analysis: H&E, Masson’s trichrome, and sirius red/fast green staining

Histological analysis was performed using standard procedures. Briefly, formalin-fixed liver was embedded in paraffin and sectioned at a thickness of 5 μm. The sections were placed on glass slides and stained with H&E, Masson’s trichrome, or Sirius red/Fast green. Histological images were captured using an Axio Imager A2 microscope (Zeiss).

### Histological analysis: CD68 immunostaining

Liver sections were heated in antigen-retrieval buffer (10 mM sodium citrate, pH 6.0, 110 °C, 20 min), quenched in 3% hydrogen peroxide/10% methanol (MeOH) (v/v) for 10 min, blocked in 10% (v/v) goat serum (catalog no.: 005-000-001; Jackson ImmunoResearch Laboratories) for 30 min, incubated with an anti-CD68 antibody (catalog no.: ab125212; abcam), held in 0.5% (v/v) goat serum in PBS (1:250 dilution) for 12 h, and incubated with a reagent from the ImmPRESS horseradish peroxidase Goat Anti-Rabbit IgG Polymer Detection Kit (catalog no.: MP-7451; Vector Laboratories) for 30 min. The ImmPACT DAB Peroxidase Substrate Kit was used for chromogenic development (catalog no.: SK-4105; Vector Laboratories). Histological images were captured using the Axio Imager A2 microscope.

### Histological analysis: immunofluorescence

For immunofluorescent staining, post-fixed liver tissues were sliced at 50 μm thickness with a microslicer (Neo-LinearSlicer MT; Dosaka EM). The tissue slices were sequentially incubated in permeabilization and blocking buffers (1% w/v Triton X-100, 2% goat serum, and 0.1% w/v sodium azide in PBS) for 24 h at room temperature. Rabbit polyclonal anti–α-SMA antibody (1:500 dilution; catalog no.: 14395-1-AP; Proteintech) was incubated for 24 h at room temperature. The tissue slices were washed three times with PBS for 10 min each and incubated with the secondary antibody (1:1000 dilution, goat anti-rabbit IgG [H + L] AlexaFluor 647–conjugated antibody [catalog no.: A-21206; Thermo Fisher Scientific]) for 24 h at room temperature. The tissue slices were washed four times with PBS for 10 min each and mounted with FluorSave Reagent (catalog no.: 345789; Millipore). Fluorescent images were captured by an SP8 LIGHTNING confocal microscope (Leica Microsystems).

### Lipid extraction and quantification

To quantify the hepatic lipid content, crude lipids were extracted from frozen liver using the Bligh–Dyer method (37). Frozen liver samples (50 mg) were homogenized in water (400 μl) using the TissueLyser LT (Qiagen) operating at 50 Hz for 5 min, and each homogenate was extracted with chloroform:MeOH 0.5:1 v/v (1.5 ml) by vortexing for 15 s. Chloroform (500 μl) and water (500 μl) were added to each homogenate, and the mixture was centrifuged (1710 × *g*, 10 min, room temperature), and the organic layer was collected. To extract more lipids, chloroform (400 μl) was added to each supernatant and admixed using a vortex mixer, followed by centrifugation (1710 × *g*, 10 min, room temperature). Each new lipid solution was combined with the original lipid solution. The combined solutions were dried under nitrogen (40 °C) and dissolved in 10% (v/v) Triton X-100 in 2-propanol (2 ml). The lipids were quantified using the Determiner-C-TG and Determiner-C-TC protocols (Minaris Medical) in terms of TG and CHO levels, respectively.

### Zymography

Gelatin and fibrinogen zymography were performed according to previously reported procedures (38). Whole-liver homogenates of frozen samples (50 mg) in ice-cold buffer (5 mM Tris–HCl, pH 7.4, 0.25 M sucrose; five volumes/tissue) were prepared using a Potter–Elvehjem device (20 strokes) and then centrifuged (13,500 × *g*, 15 min, 4°C), after which the supernatants were collected. Protein levels were quantified using a BCA assay kit (catalog no.: 23225; Thermo Fisher Scientific), and proteins (20 μg/lane) were separated *via* SDS-PAGE (with 2 mg/ml fibrinogen or 1 mg/ml gelatin). The gels were washed twice with 2.5% (v/v) Triton X-100 for 30 min at room temperature and incubated with gelatin- or fibrinogen-containing buffers for 24 or 48 h, respectively, prior to zymography in 25 mM Tris–HCl (pH 7.5), 15 mM NaCl, 5 mM CaCl_2_, and 10 μM ZnCl_2_ or in 50 mM Tris–HCl (pH 8.5) and 0.1 M glycine. The gels were stained with Coomassie Brilliant Blue and destained with 5% (v/v) MeOH/7% (v/v) acetic acid. Gel images were captured using the ChemiDoc MP imaging system (Bio-Rad).

### RNA extraction and RT–qPCR analysis

Total RNA was extracted from frozen liver using RNAiso Plus (catalog no.: 9109; TaKaRa Bio) and TissueLyser LT (50 Hz, 3 min). The RNA concentration was measured using a Nanodrop Lite (Thermo Fisher Scientific). Extracted total RNA was transcribed to complementary DNA using a PrimeScript RT Reagent Kit (catalog no.: RR037B; TaKaRa Bio), and mRNA levels were measured using KOD SYBR qPCR Mix (catalog no.: QKD-201; Toyobo) on an Applied Biosystems StepOne Realtime PCR System (Thermo Fisher Scientific) or a CFX Connect Realtime PCR System (Bio-Rad) with the delta–delta-Ct method. Rn18s and Gapdh were used as internal standards. Primer sequences are listed in Table S1.

### RNA extraction and sequencing

Total RNA was extracted from frozen liver samples using the RNeasy Mini kit (catalog no.: 74104; Qiagen). After RNA quantification and quality control, libraries were constructed using the TruSeq Stranded mRNA LT Sample Prep kit (Illumina) according to the manufacturer’s protocol (catalog no.: 15031047; Rev. E). The NovaSeq 6000 (Illumina) sequence paired-end read length was set to 2 × 100 bp; raw data were processed and checked using the Galaxy (version 20.01) system. FastQC, version 0.72, was used for adapter trimming and quality filtering; fastp, version 0.19.5, for alignment; and STAR, version 2.7.1a, for rRNA removal followed by application of bedtools (in the intersect mode), version 2.29.0; and featurecounts, version 1.6.4, for quantification. The filtered data were mapped onto the Gencode reference genome (GRCm38.p6) and annotated using Gencode (M24). Multivariate analysis, normalization, differentially expressed gene identification, and data visualization were performed using R software (R Foundation for Statistical Computing). Read counts were normalized using DESeq2 (39); differentially expressed genes were identified using a combination of DESeq2 and EBSeqHMM (40). Gene set enrichment analysis was performed using clusterProfiler (41), and gene sets (or pathways) were selected using MSigDB Hallmark (42) and KEGG (Kyoto Encyclopedia of Genes and Genomes) (43). All human MASLD–MASH transcriptome signature genes were curated manually from a prior report (18).

### Untargeted metabolomic analysis using GC–MS

Untargeted metabolomic analysis was performed by GC–MS (44). Data analysis was performed using MS-DIAL (version 3.70; RIKEN PRIMe) (45) and MetaboAnalyst 4.0 online tool (https://www.metaboanalyst.ca/) (46). Frozen liver tissue samples (50 mg) were homogenized in 1 ml of MeOH/chloroform/water (2.5:1:1 v/v/v), and 10 μl of an internal standard solution (1.2 mg/ml Adonitol [catalog no.: A5502; Sigma–Aldrich] in water) was added to each homogenate. The homogenates were vigorously mixed on a temperature-controlled shaker (catalog no.: MBR-022UP; TAITEC) for 30 min at 20 °C; centrifuged at 16,000 × *g* for 5 min at 4 °C; and water (200 μl) added to each supernatant, followed by centrifugation (20,000 × *g*, 15 min, 4 °C). Each upper layer (600 μl) was collected; snap frozen in liquid nitrogen; and dried in a centrifugal concentrator for 16 h. Prior to TMS derivatization, 100 μl amounts of 20 mg/ml methoxyamine hydrochloride (catalog no.: 226904; Sigma–Aldrich)/pyridine were added to each sample, followed by sonication twice for 10 s. The samples were then placed in a shaker for 90 min at 30 °C; 50 μl *N*-methyl-*N*-trimethylsilyl-trifluoroacetamide (catalog no.: 69479; Sigma–Aldrich) was added to each sample; followed by incubation in a shaker for 30 min at 30 °C and GC–MS (Agilent 7890B GC, Agilent Technologies; JEOL JMS-Q1500GC, JEOL) analysis. The samples (1 μl) were injected at a 25:1 split ratio, and the analytes separated on an InertCap 5MS/NP column (30 m, 250 μm internal diameter, 0.25 μm film thickness; catalog no.: 1010-18642; GL Science) using helium as the carrier gas (1.120 ml/min). The column oven temperature was held at 80 °C for 2 min, then increased to 330 °C (at 15 °C/min), and held for 9 min. The other analytical conditions were as follows: injector temperature, 250 °C; septum purge flow, 5 ml/min; solvent delay, 3.5 min; MS ion source temperature, 200 °C; MS interface temperature, 250 °C; and MS scan mode, 85 to 500 *m/z*. A saturated hydrocarbon standard mixture (catalog no.: 1021-58321 GL Science) was used to adjust the retention time drifts. The retention index was based on the alkane retention times. Spectral deconvolution and peak identification were performed with the aid of MS-DIAL software and a reference database (GL-Science DB Kovats retention index for the InertCap 5MS/NP column). Multivariate analysis and data visualization employed R software. Pathway analysis was performed with the aid of MetaboAnalyst.

### Liver Hyp quantification

Hepatic Hyp was quantified by LC–MS/MS. Frozen liver samples (30–60 mg) were weighed, freeze-dried for 16 h, weighed again, and hydrolyzed in 6 M HCl (with 0.4% [v/v] 3-mercapto-1,2-propanediol [10 mg dry weight/ml]) for 3 h at 110 °C. After cooling, the samples were centrifuged (20,000 × *g*, 10 min, room temperature) and the supernatants filtered through a hydrophilic polyvinylidene fluoride (PVDF) filter (0.2 μm pore size; catalog no.: SLLGX13NL; Millipore). The filtered hydrolysates (15 μl) were mixed with 6 M HCl (75 μl) and acetonitrile (ACN; 810 μl) and subjected to LC–MS/MS (Acquity UPLC platform, Quattro Premier XE; Waters). To derive a calibration curve, Hyp (catalog no.: H0296; Tokyo Chemical Industry) was dissolved in 6 M HCl and serially diluted. Chromatographic separation employed an Intrada Amino Acid column (100 mm × 3 mm internal diameter, 3-μm particle size, catalog no.: WAA34; Imtakt). Mobile phase A was 100 mM ammonium formate in water, and mobile phase B was 0.3% v/v formic acid in ACN. The gradients were 0 to 0.5 min, 90% B; to 3.5 min, 70% B; to 5 to 6 min, 0% B; to 6.6 min, 90% B; and to 8 min, 90% B hold. The flow rates were 0.6 ml/min (0–5 min and 7.6–8 min) and 0.7 ml/min (5.1–7.5 min); the column temperature was 40 °C; the injection volume was 2 μl; and the monitoring ion was *m/z* 132.0 > 68.0 (electrospray ionization positive; cone voltage 20 V, and collision energy 15 V). The Hyp levels were divided by the wet tissue weights.

### Targeted quantification of liver metabolites using LC–MS/MS

Liver metabolites were extracted as described previously (47). Targeted metabolite quantification was performed by LC–MS/MS. Frozen liver samples (50 mg) were homogenized in extraction of solvent A (460 μl, ACN/MeOH/water [40/40/20 v/v/v] with 0.1 M formic acid) using the TissueLyzer LT (50 Hz, 3 min). For neutralization, extraction of solvent B (40 μl; 15% [w/v] ammonium bicarbonate in water) was added to each homogenate. After centrifugation (20,000 × *g*, 5 min, 4 °C), the supernatants (195 μl) were mixed with 5 μl of an internal standard solution (200 μM norvaline [catalog no.: N0304; Tokyo Chemical Industry] and 2-(*N*-morpholino) ethanesulfonic acid [catalog no.: M3671; Sigma–Aldrich] in water). The standard compounds were dissolved in water or 0.1 M formic acid to 10 to 20 mM and then further diluted in neutralized extraction solvent (solvent A:B = 100:8.7 μl) to obtain calibration curves. Liver metabolites were quantified using the LC–MS/MS system described above. Chromatographic separation was performed using a Triart-PFP column (150 mm × 2.1 mm ID; 3 μm diameter particles, catalog no.: TPF12S03-15Q1PTH; YMC). Mobile phases A and B were 0.1% (v/v) formic acid in water and 0.1% (v/v) formic acid in ACN, respectively. The gradient was as follows: 0% B from 0 to 2 min, 10% B to 4 min, 40% B to 7 min, 100% B to 8 min, 100% B hold to 11 min, and 0% B to 15 min. The flow rate was 0.2 ml/min, the column temperature was 40 °C, and the injection volume was 2 μl. The electrospray ionization mode, monitoring ion, declustering potential, and collision energy are listed in Table S2. Data were quantified using MassLynx software (Waters) and visualized using R software.

### Blood biochemistry and amino acid analysis

The ALT and AST levels in plasma were measured using a chemical analyzer (DRI-CHEM 4000V; Fujifilm). The amino acid concentrations in blood plasma were quantified with the LC–MS/MS system used for liver metabolite quantification. Derivatization and analysis were performed with the AccQ-Tag Ultra Derivatization Kit (catalog no.: 186003836; Waters) and an AccQ-Tag Ultra RP column (100 mm × 2.1 mm ID, 1.7 μm particle size; catalog no.: 186003837; Waters) according to the manufacturer’s instructions. The amino acid standards were dissolved in 0.1 M HCl and diluted to prepare the calibration curve. Norvaline was used as the internal standard.

### Western blotting

Western blotting was performed according to previously reported procedures (41). Total histones were extracted from frozen liver samples (100 mg) using the EpiQuik Total Histone Extraction Kit (catalog no.: OP-0006-100; EpiGentek) according to the manufacturer’s instructions. The histones were separated *via* electrophoresis (3 μg/lane) and transferred to PVDF membranes (Immobilon-P PVDF membrane; catalog no.: IPVH00010; Millipore). The membranes were blocked with the PVDF Blocking Reagent for the Can-Get Signal (catalog no.: NYPBR01; Toyobo) and incubated with the following primary antibodies for 16 h at 4 °C: rabbit anti-H3K4me3 (1:1000 dilution, catalog no.: 9751; Cell Signaling Technology), rabbit anti-H3K9me3 (1:1000 dilution, catalog no.: 13969; Cell Signaling Technology), rabbit anti-H3K27me3 (1:1000 dilution, catalog no.: 9733; Cell Signaling Technology), rabbit anti-H3K36me3 (1:100 dilution, catalog no.: 4909; Cell Signaling Technology), rabbit anti-H3K79me3 (1:1000 dilution, catalog no.: 4260; Cell Signaling Technology), and rabbit anti-histone H3 (1:1000 dilution, catalog no.: 7168-1-AP; Proteintech). The primary antibodies were diluted with Can-Get Signal Immunoreaction Enhancer Solution 1 (catalog no.: NKB-201; Toyobo). After three washes (5 min each) with 0.05% (v/v) Tween-20/Tris-buffered saline, the membranes were incubated with horseradish peroxidase–conjugated goat anti-rabbit antibody (1:20,000 dilution, catalog no.: 111-035-003; Jackson ImmunoResearch Laboratories) for 1 h. The secondary antibodies were diluted in 1% (w/v) nonfat dry milk in PBS. The membranes were washed three times for 5 min each and developed using the ImmunoStar LD reagent (catalog no.: 290-69904; Fujifilm Wako Pure Chemical). Chemiluminescence was captured by the ChemiDoc MP imaging system (Bio-Rad). Densitometric analysis was performed using ImageJ software (48).

### Statistical analysis

Statistical analysis was performed using R software and Prism 10 (GraphPad Software, Inc). For comparisons of two groups, the two-tailed unpaired Welch’s *t* test was used. For comparisons of more than two groups, Tukey’s test was used. A *p* value < 0.05 was considered to denote statistical significance, unless otherwise indicated.

## Data availability

Any additional information required to reanalyze the data reported in this article is available from the lead contact upon request. RNA-Seq raw data were deposited in the public database DNA Data Bank of Japan (DDBJ) Sequence Read Archive (DRA, accession number: DRA015440). RNA-Seq count data were deposited in the public database DDBJ Genomic Expression Archive (accession number: E-GEAD-819). Metabolomics data were deposited in the public database DDBJ MetaboBank database (accession number: MTBKS254).

## Supporting information

This article contains supporting information.

## CRediT authorship contribution statement

**Atsushi Miura:** Writing – original draft, Investigation, Data curation, Methodology, Funding acquisition, Conceptualization, Writing – review & editing, Visualization. **Shiori Ikeda:** Investigation. **Yuki Kono:** Investigation. **Keigo Kawate:** Investigation. **Takashi Hosono:** Project administration, Writing – review & editing. **Taiichiro Seki:** Funding acquisition, Writing – review & editing.

## Conflict of interest

The authors declare that they have no conflicts of interest with the contents of this article.
