# Supplementary material for: Metabolic and epigenetic abnormalities cause hepatic fibrogenesis in metabolic dysfunction–associated steatohepatitis model mice
Source: J Biol Chem. 2025 Nov 20;302(1):110959. doi: 10.1016/j.jbc.2025.110959 (PMC12775946; doi:10.1016/j.jbc.2025.110959)
Supplement: Supplementary Tables [file mmc1.docx]

**Supporting Tables**

| **Table S1. Primer pairs used for qPCR.** | | |
| --- | --- | --- |
| *Gene symbol* | Forward (5’ to 3’) | Reverse (5’ to 3’) |
| *Tgfb1* | CGCAACAACGCCATCTATGA | ACTGCTTCCCGAATGTCTGA |
| *Col1a1* | GCTCCTCTTAGGGGCCACT | CCACGTCTCACCATTGGGG |
| *Col3a1* | ACGTAAGCACTGGTGGACAG | CAGGAGGGCCATAGCTGAAC |
| *Acta2* | AAACAGGAATACGACGAA | CAGGAATGATTTGGAAAGGA |
| *Ctgf* | GGGCCTCTTCTGCGATTTC | ATCCAGGCAAGTGCATTGGTA |
| *Bambi* | GATCGCCACTCCAGCTACTTC | GCAGGCACTAAGCTCAGACTT |
| *F4/80 (Adgre1)* | CCCCAGTGTCCTTACAGAGTG | GTGCCCAGAGTGGATGTCT |
| *Trem2* | CTGGAACCGTCACCATCACTC | CGAAACTCGATGACTCCTCGG |
| *Ccl2* | CTTCTGGGCCTGCTGTTCA | CCAGCCTACTCATTGGGATCA |
| *Gpnmb* | TCTGAACCGAGCCCTGACATC | AGCAGTAGCGGCCATGTGAAG |
| *Tnfa* | GAGAAAGTCAACCTCCTCTCTG | GAAGACTCCTCCCAGGTATATG |
| *Il6* | GCAAGAGACTTCCATCCAGTTGC | AAGTCTCCTCTCCGGACTTGTG |
| *Mmp9* | GCCGACTTTTGTGGTCTTCC | CTTCTCTCCCATCATCTGGGC |
| *Mmp2* | CCCCATGAAGCCTTGTTTACC | TTGTAGGAGGTGCCCTGGAA |
| *Mmp13* | TCCCTGGAATTGGCAACAAAG | GCATGACTCTCACAATGCGATTAC |
| *Timp1* | GCATCTCTGGCATCTGGCATC | GCGGTTCTGGGACTTGTGGGC |
| *Plat* | TTCTGGCTACGGCAAGCATG | CGCACAGCATGTTGTTCGTG |
| *Plau* | TGGAGCAGCTCATCTTGCAC | TGGAGCAGCTCATCTTGCAC |
| *Serpine1* | ACGCCTGGTGCTGGTGAATGC | ACGGTGCTGCCATCAGACTTGTG |
| *G6pdx* | CACAGTGGACGACATCCGAAA | AGCTACATAGGAATTACGGGCAA |
| *Rn18s* | GTAACCCGTTGAACCCCATT | CCATCCAATCGGTAGTAGCG |
| *Gapdh* | TGTGTCCGTCGTGGATCTG | GAGACAACCTGGTCCTCAGTG |

| **Table S2. Analytical settings used for LC-MS/MS metabolite quantification.** | | | | | |
| --- | --- | --- | --- | --- | --- |
| **Compound** | **Precursor ion** | **Product ion** | **Cone (V)** | **CE (V)** | **ESI mode** |
| Glutathione | 308.1 | 75.7 | 20 | 25 | positive |
| Glutathione disulfide | 613.2 | 230.8 | 30 | 35 | positive |
| *S*-adenosylmethionine | 400.1 | 250.8 | 20 | 15 | positive |
| *S*-adenosylhomocysteine | 385.1 | 133.6 | 25 | 25 | positive |
| Methionine | 150.1 | 55.7 | 15 | 15 | positive |
| 5'-Methylthioadenosine | 298.1 | 135.6 | 20 | 20 | positive |
| Homocysteine | 136 | 55.7 | 20 | 20 | positive |
| Phosphocreatine | 212 | 43.9 | 20 | 20 | positive |
| Argininosuccinate | 291.1 | 69.8 | 25 | 30 | positive |
| Carbamoyl phosphate | 140 | 96.6 | 10 | 10 | negative |
| *N*-Acetylglutamate | 190.1 | 83.7 | 15 | 25 | positive |
| Choline | 105.1 | 58.9 | 35 | 20 | positive |
| *N,N,N*-Trimethylglycine | 118.1 | 57.8 | 30 | 25 | positive |
| *N,N*-Dimethylglycine | 104.1 | 57.8 | 20 | 15 | positive |
| L-Carnitine | 162.1 | 42.9 | 30 | 20 | positive |
| Creatinine | 114.1 | 43.9 | 25 | 15 | positive |
| Urea | 61 | 43.9 | 20 | 10 | positive |
| Spermine | 203.2 | 111.8 | 20 | 20 | positive |
| Spermidine | 146.2 | 71.8 | 20 | 15 | positive |
| 2-(*N*-Morpholino) ethanesulfonic acid (MES, IS) | 196.1 | 99.7 | 35 | 20 | positive |
| Norvaline (IS) | 118.1 | 71.6 | 15 | 10 | positive |
| Cycloleucine | 130.1 | 83.6 | 20 | 15 | positive |
