# Supplementary material for: Metabolic and epigenetic abnormalities cause hepatic fibrogenesis in metabolic dysfunction–associated steatohepatitis model mice
Source: J Biol Chem. 2025 Nov 20;302(1):110959. doi: 10.1016/j.jbc.2025.110959 (PMC12775946; doi:10.1016/j.jbc.2025.110959)
Supplement: Supplementary Figures [file mmc2.pdf]

Supporting information

Metabolic and Epigenetic Abnormalities Cause Hepatic Fibrogenesis in Metabolic Dysfunction-Associated Steatohepatitis Model Mice

Supplementary Figure 1
Supplementary Figure 2
Supplementary Figure 3
Supplementary Figure 4

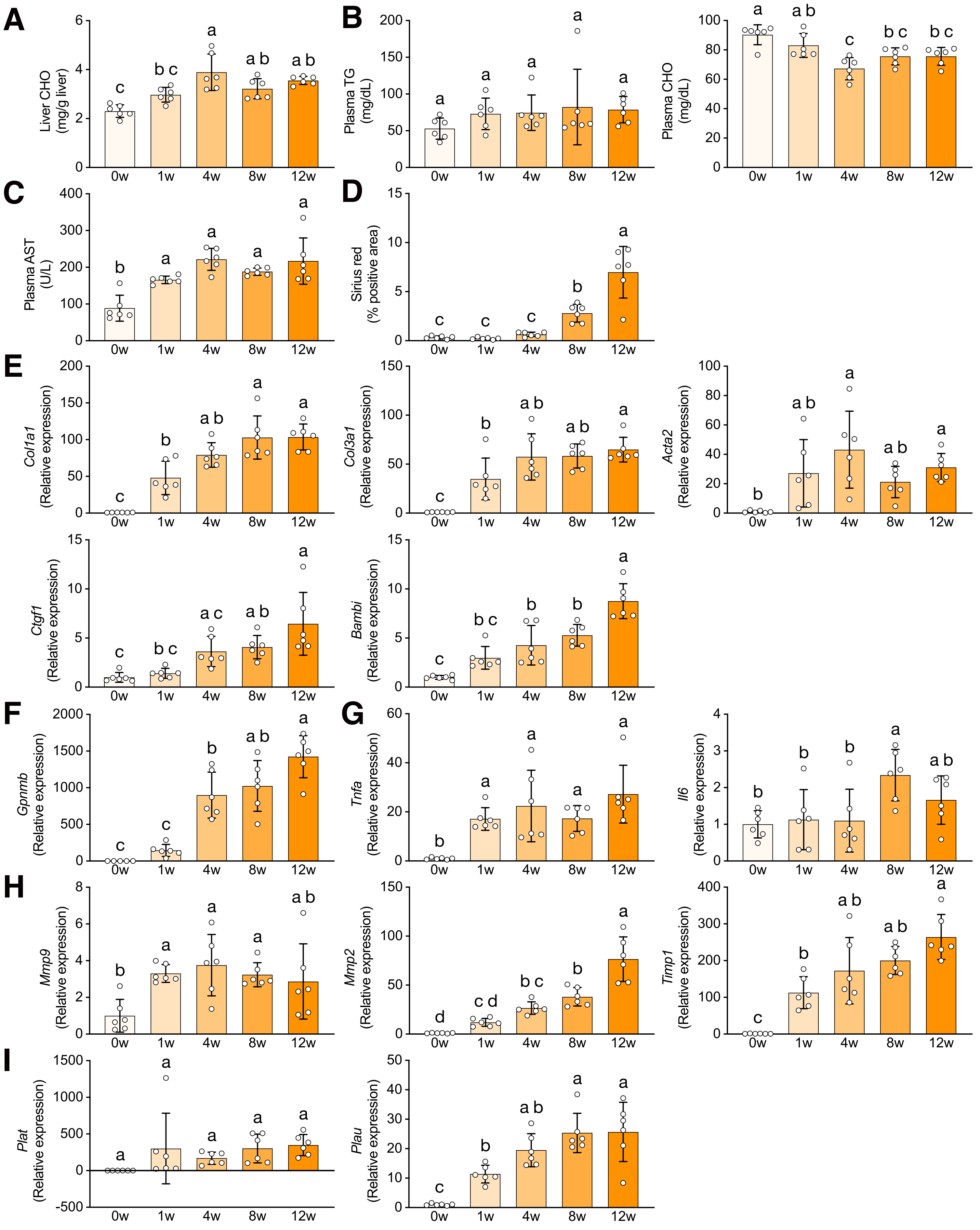
A
Liver CHO
(mg/g liver)
0w
1w
4w
8w
12w
B
Plasma TG
(mg/dL)
Plasma CHO
(mg/dL)
C
Plasma AST
(U/L)
D
Sirius red
(% positive area)
E
Col1a1
(Relative expression)
Col3a1
(Relative expression)
Acta2
(Relative expression)
Ctgf1
(Relative expression)
Bambi
(Relative expression)
F
Gpnmb
(Relative expression)
G
Tnfa
(Relative expression)
Il6
(Relative expression)
H
Mmp9
(Relative expression)
Mmp2
(Relative expression)
Timp1
(Relative expression)
I
Plat
(Relative expression)
Plau
(Relative expression)

Fig. S1l Changes in plasma and liver biochemistry, histology, and gene expression over time in the CDAHFD-fed mouse liver. (A) Hepatic CHO content. (B) Plasma TG and CHO levels. (C) Plasma aspartate aminotransferase (AST) level. (D) Image analysis of Sirius red-stained sections using ImageJ. (E-I) Hepatic gene expression. Data are means ± SD (n = 6). Different letters indicate significant differences by Tukey’ s test, α = 0.05.

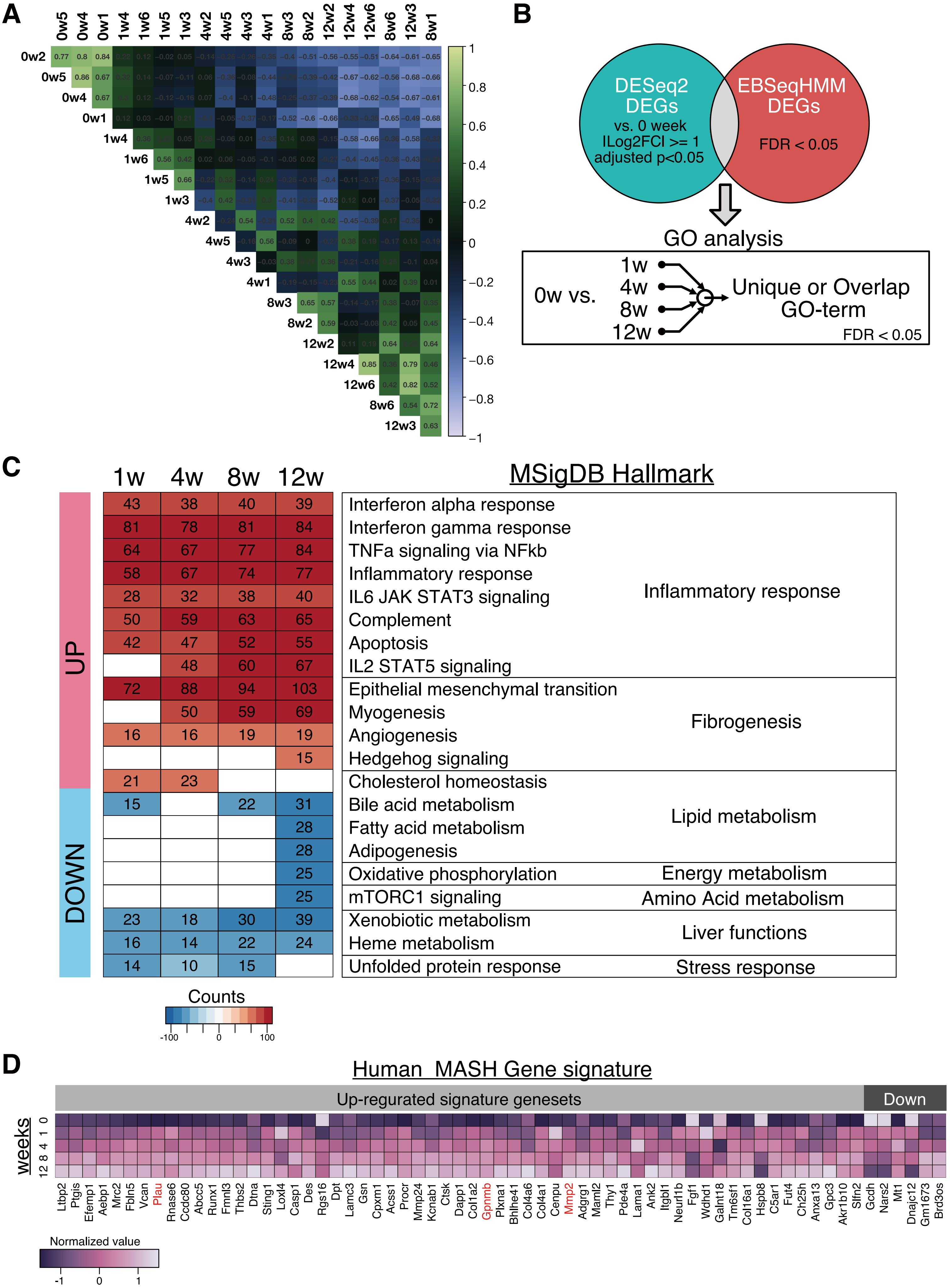
A
0w5 0w4 0w1 1w4 1w6 1w5 1w3 4w2 4w5 4w3 4w1 8w3 8w2 12w2 12w4 12w6 8w6 12w3 8w1
0w2 0w5 0w4 0w1 1w4 1w6 1w5 1w3 4w2 4w5 4w3 4w1 8w3 8w2 12w2 12w4 12w6 8w6 12w3
1 0.8 0.6 0.4 0.2 0 −0.2 −0.4 −0.6 −0.8 −1
B
DESeq2 DEGs
vs. 0 week
lLog2FCl >= 1
adjusted p<0.05
EBSeqHMM DEGs
FDR < 0.05
GO analysis
0w vs.
1w
4w
8w
12w
Unique or Overlap GO-term
FDR < 0.05
C
1w 4w 8w 12w
MSigDB Hallmark
UP
DOWN
43 38 40 39 Interferon alpha response
81 78 81 84 Interferon gamma response
64 67 77 84 TNFa signaling via NFkb
58 67 74 77 Inflammatory response
28 32 38 40 IL6 JAK STAT3 signaling
50 59 63 65 Complement
42 47 52 55 Apoptosis
48 60 67 IL2 STAT5 signaling
Inflammatory response
72 88 94 103 Epithelial mesenchymal transition
50 59 69 Myogenesis
16 16 19 19 Angiogenesis
15 Hedgehog signaling
Fibrogenesis
21 23 Cholesterol homeostasis
15 22 31 Bile acid metabolism
28 Fatty acid metabolism
28 Adipogenesis
Lipid metabolism
25 Oxidative phosphorylation
Energy metabolism
25 mTORC1 signaling
Amino Acid metabolism
23 18 30 39 Xenobiotic metabolism
16 14 22 24 Heme metabolism
Liver functions
14 10 15 Unfolded protein response
Stress response
Counts
-100 0 100
D
Human MASH Gene signature
Up-regurated signature genesets
Down
weeks
0 1 4 8 12
Ltbp2 Ptgis Efemp1 Aebp1 Mrc2 Fbln5 Vcan Plau Rnase6 Ccdc80 Abcc5 Runx1 Fmnl3 Thbs2 Dtna Sting1 Loxl4 Casp1 Des Rgs16 Dpt Lamc3 Gsn Cpxm1 Acss1 Procr Mmp24 Kcnab1 Ctsk Dapp1 Col1a2 Gpnmb Plxna1 Bhlhe41 Col4a6 Col4a1 Cenpu Mmp2 Adgrg1 Maml2 Thy1 Pde4a Lama1 Ank2 Itgbl1 Neurl1b Fgf1 Wdhd1 Galnt18 Tm6sf1 Col16a1 Hspb8 C5ar1 Fut4 Ch25h Anxa13 Gpc3 Akr1b10 Slfn2 Gcdh Nars2 Mt1 Dnajc12 Gm1673 Brd3os
Normalized value
-1 0 1

Fig. S2I Transcriptomic analysis of the CDAHFD-fed mouse liver. (A) Correlation matrix of liver transcriptome analyzed by RNA-seq. (B) Statistical criteria for Gene Ontology (GO)-term selection. DESeq2 and EBSeqHMM were used to identify differentially expressed genes (DEGs). GO analysis (GSEA) was performed using clusterProfiler. (C) Enrichment analysis. DEGs were mapped onto the MSigDB gene set. (D) Heatmap of the human MASH gene signature. The CDAHFD liver transcriptome was integrated with reported gene signatures of human MASH. Red, genes verified by qPCR.

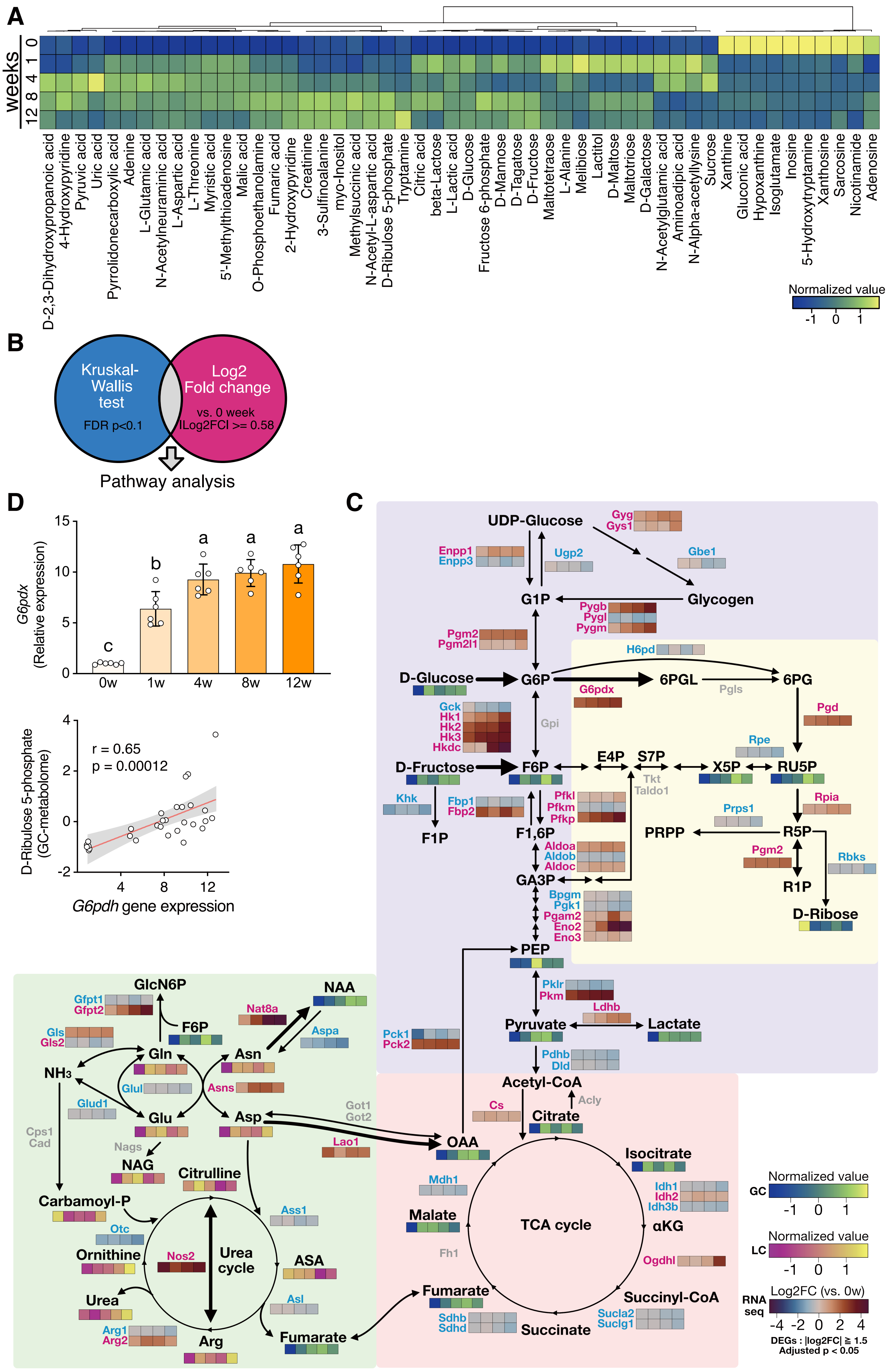
A
weeks
0
1
4
8
12
D-2,3-Dihydroxypropanoic acid
4-Hydroxypyridine
Pyruvic acid
Uric acid
Pyrrolidonecarboxylic acid
Adenine
L-Glutamic acid
N-Acetylneuraminic acid
L-Aspartic acid
L-Threonine
Myristic acid
5'-Methylthioadenosine
Malic acid
O-Phosphoethanolamine
Fumaric acid
2-Hydroxypyridine
Creatinine
3-Sulfinoalanine
myo-Inositol
Methylsuccinic acid
N-Acetyl-L-aspartic acid
D-Ribulose 5-phosphate
Tryptamine
Citric acid
beta-Lactose
L-Lactic acid
D-Glucose
Fructose 6-phosphate
D-Mannose
D-Tagatose
D-Fructose
Maltotetraose
L-Alanine
Melibiose
Lactitol
D-Maltose
Maltotriose
D-Galactose
N-Acetylglutamic acid
Aminoadipic acid
N-Alpha-acetyllysine
Sucrose
Xanthine
Gluconic acid
Hypoxanthine
Isoglutamate
Inosine
5-Hydroxytryptamine
Xanthosine
Sarcosine
Nicotinamide
Adenosine
Normalized value
-1 0 1
B
Kruskal-Wallis test
FDR p<0.1
Log2 Fold change
vs. 0 week
|Log2FC| >= 0.58
Pathway analysis
D
G6pdx (Relative expression)
c b a a a
0w 1w 4w 8w 12w
D-Ribulose 5-phosphate (GC-metabolome)
r = 0.65
p = 0.00012
G6pdh gene expression
C
UDP-Glucose
Gyg
Gys1
Enpp1
Enpp3
Ugp2
Gbe1
G1P
Glycogen
Pygb
Pygl
Pygm
Pgm2
Pgm2l1
H6pd
D-Glucose
G6P
6PGL
Pgls
6PG
G6pdx
Gck
Hk1
Hk2
Hk3
Hkdc
Gpi
Pgd
Rpe
D-Fructose
F6P
E4P
S7P
X5P
RU5P
Tkt
Taldo1
Khk
Fbp1
Fbp2
Pfkl
Pfkm
Pfkp
Rpia
Prps1
F1P
F1,6P
PRPP
R5P
Aldoa
Aldob
Aldoc
Pgm2
Rbks
GA3P
R1P
Bpgm
Pgk1
Pgam2
Eno2
Eno3
D-Ribose
PEP
Pklr
Pkm
Ldhb
Pck1
Pck2
Pyruvate
Lactate
Pdhb
Dld
Acetyl-CoA
Acly
Cs
Citrate
GlcN6P
NAA
Gfpt1
Gfpt2
Nat8a
F6P
Aspa
Gls
Gls2
Gln
Asn
NH3
Glul
Asns
Glud1
Glu
Asp
Got1
Got2
Cps1
Cad
Nags
Lao1
OAA
NAG
Citrulline
Isocitrate
Mdh1
Idh1
Idh2
Idh3b
Carbamoyl-P
Ass1
Malate
TCA cycle
αKG
Otc
Ornithine
Nos2
Urea cycle
ASA
Fh1
Ogdhl
Urea
Asl
Fumarate
Succinyl-CoA
Arg1
Arg2
Sdhb
Sdhd
Sucla2
Suclg1
Arg
Fumarate
Succinate
Normalized value
GC
Normalized value
LC
RNA seq
Log2FC (vs. 0w)
-4 -2 0 2 4
DEGs : |log2FC| ≧ 1.5
Adjusted p < 0.05

Fig. S3I Untargeted metabolomic analysis of the CDAHFD-fed mouse liver. (A) Heatmap of the metabolites that changed over time in the liver. Metabolite levels were measured by GC/MS. (B) Statistical criteria for metabolite selection. Kruskal-Wallis test and log2FC were combined to identify metabolites that changed over time. (C) Curated metabolic pathways. The carbohydrate anabolic/catabolic pathways, pentose phosphate pathway, tricarboxylic acid cycle (TCA) cycle, and urea cycle-related pathway are shown in different colors. Red, upregulated gene; blue, down-regulated gene; gray, not significant. (D) Hepatic gene expression of *G6pdx* and its correlation with the Ribu-5P level. Data are means ± SD (n = 5 - 6). Different letters indicate significant differences by Tukey's test, $\alpha = 0.05$.

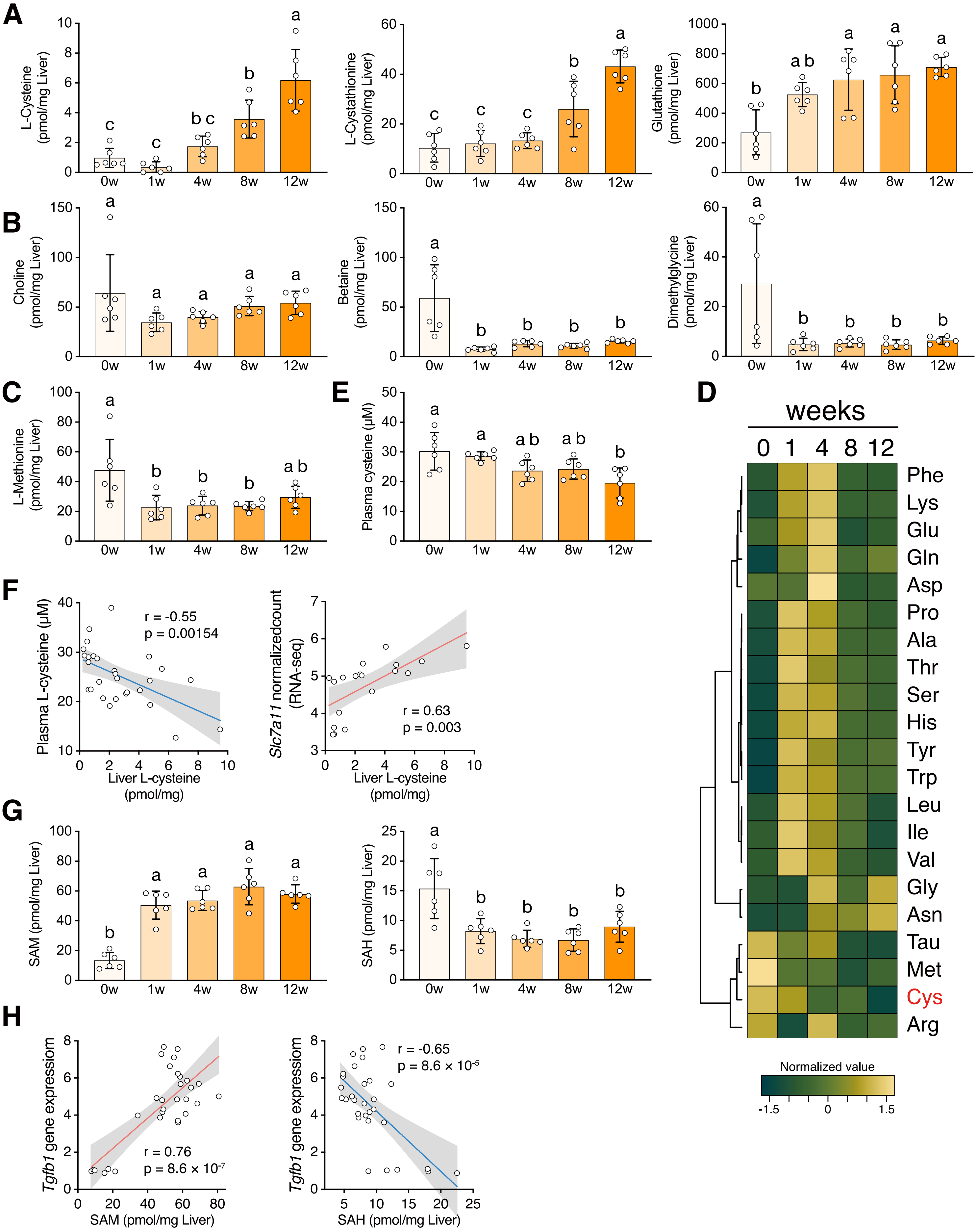

A
L-Cysteine (pmol/mg Liver)
L-Cystathionine (pmol/mg Liver)
Glutathione (pmol/mg Liver)
0w 1w 4w 8w 12w
B
Choline (pmol/mg Liver)
Betaine (pmol/mg Liver)
Dimethylglycine (pmol/mg Liver)
C
L-Methionine (pmol/mg Liver)
E
Plasma cysteine (μM)
D
weeks
0 1 4 8 12
Phe
Lys
Glu
Gln
Asp
Pro
Ala
Thr
Ser
His
Tyr
Trp
Leu
Ile
Val
Gly
Asn
Tau
Met
Cys
Arg
Normalized value
-1.5 0 1.5
F
Plasma L-cysteine (μM)
r = -0.55
p = 0.00154
Liver L-cysteine (pmol/mg)
Slc7a11 normalizedcount (RNA-seq)
r = 0.63
p = 0.003
G
SAM (pmol/mg Liver)
SAH (pmol/mg Liver)
H
Tgfb1 gene expressiom
r = 0.76
p = 8.6 × 10-7
SAM (pmol/mg Liver)
Tgfb1 gene expression
r = -0.65
p = 8.6 × 10-5
SAH (pmol/mg Liver)

Fig. S4I Methionine-SAM-cysteine metabolite analysis in the CDAHFD-fed mice. (A-C) Hepatic metabolite levels. (D) Heatmap of the plasma amino acid profile. (E) Plasma Cys level. (F) Correlations of the hepatic and plasma Cys levels with hepatic *Slc7a11* gene expression. (G) Hepatic SAM and SAH levels. (H) Correlation of SAM and SAH levels with *Tgfb1* gene expression in the liver. Data are means ± SD ($n = 6$). Different letters indicate significant differences by Tukey's test, $\alpha = 0.05$.
